# Supplementary material for: Evaluating Desk-Assisted Standing Techniques for Simulated Pregnant Conditions: An Experimental Study Using a Maternity-Simulation Jacket
Source: Healthcare (Basel). 2024 May 1;12(9):931. doi: 10.3390/healthcare12090931 (PMC11083540; doi:10.3390/healthcare12090931)
Supplement: Supplementary file 1 [file healthcare-12-00931-s001.zip › healthcare-2928706-supplementary.pdf]

Supplement "Evaluating Desk-Assisted Standing Techniques for Simulated Pregnant Conditions: An Experimental Study Using a Maternity-Simulation Jacket"

To supplement the manuscript, we present the results not only in graphs but also in tables.

1. Comparison of muscle load during STS using EMG analysis

In figure 4, we compare left and right EMG. Table S1 and Table S2 show the details.

Table S1. iEMG of left and right lower back.

conditions	Mean	SD
**both-hands-on-knees**		
left	485.07	176.81
right	440.39	230.48
**one-hand-on-knees**		
left	453.62	232.12
right	525.60	267.41
**both-hands-on-tables**		
left	545.25	222.08
right	482.97	238.46

Table S2. maximum EMG of left and right lower back.

conditions	Mean	SD
**both-hands-on-knees**		
left	43.58	13.95
right	37.62	18.77
**one-hand-on-knees**		
left	47.25	22.36
right	48.89	23.99
**both-hands-on-tables**		
left	42.83	18.29
right	33.61	18.23

2. *Comparison of trunk flexion angle during STS*

In figure 5b, we compare the maximum flexion angle. Table S3 shows the details.

Table S3. maximum trunk flexion angles.

conditions	Mean	SD
both-hands-on-knees	33.00	4.85
one-hand-on-knees	25.87	8.65
both-hands-on-tables	34.58	3.76

3. *Reproducing the pregnant state*

In figure 6, we compare the results wearing the jacket and not wearing the jacket. Table S4, S5, S6, and S7 provide the details.

Table S4. iEMG wearing the jacket and not wearing the jacket.

conditions	Mean	SD
**both-hands-on-knees**		
wearing the jacket	462.73	204.70
not wearing the jacket	295.70	144.64
**one-hand-on-knees**		
wearing the jacket	489.61	250.66
not wearing the jacket	328.82	229.06
**both-hands-on-tables**		
wearing the jacket	514.11	229.29
not wearing the jacket	371.77	192.66

Table S5. Maximum EMG wearing the jacket and not wearing the jacket.

conditions	Mean	SD
**both-hands-on-knees**		
wearing the jacket	40.60	16.65
not wearing the jacket	33.24	13.14
**one-hand-on-knees**		
wearing the jacket	48.07	22.99
not wearing the jacket	32.32	19.43
**both-hands-on-tables**		
wearing the jacket	38.22	18.59
not wearing the jacket	32.06	13.81

Table S6. Y-axis mean of COP wearing the jacket and not wearing the jacket.

conditions	Mean	SD
**both-hands-on-knees**		
wearing the jacket	-59.80	15.14
not wearing the jacket	-42.12	20.14
**one-hand-on-knees**		
wearing the jacket	-56.14	11.45
not wearing the jacket	-37.64	22.25
**both-hands-on-tables**		
wearing the jacket	-52.79	22.28
not wearing the jacket	-35.80	19.96

Table S7. maximum trunk flexion angles wearing the jacket and not wearing the jacket.

conditions	Mean	SD
**both-hands-on-knees**		
wearing the jacket	33.00	4.85
not wearing the jacket	35.54	6.52
**one-hand-on-knees**		
wearing the jacket	25.87	8.65
not wearing the jacket	29.35	8.86
**both-hands-on-tables**		
wearing the jacket	34.58	3.76
not wearing the jacket	34.43	6.43
